# Depression, Inflammation, and Intestinal Permeability: Associations with Subjective and Objective Cognitive Functioning throughout Breast Cancer Survivorship

**DOI:** 10.3390/cancers15174414

**Published:** 2023-09-04

**Authors:** Annelise A. Madison, Rebecca Andridge, Anthony H. Kantaras, Megan E. Renna, Jeanette M. Bennett, Catherine M. Alfano, Stephen P. Povoski, Doreen M. Agnese, Maryam Lustberg, Robert Wesolowski, William E. Carson, Nicole O. Williams, Raquel E. Reinbolt, Sagar D. Sardesai, Anne M. Noonan, Daniel G. Stover, Mathew A. Cherian, William B. Malarkey, Janice K. Kiecolt-Glaser

**Affiliations:** 1 Institute for Behavioral Medicine Research, The Ohio State University College of Medicine, The Ohio State University, Columbus, OH 43210, USA; andridge.1@osu.edu (R.A.); william.malarkey@osumc.edu (W.B.M.); kiecolt-glaser.1@osu.edu (J.K.K.-G.); 2 Department of Psychology, The Ohio State University, Columbus, OH 43210, USA; 3 Division of Biostatistics, The Ohio State University, Columbus, OH 43210, USA; 4 Department of Neuroscience, The Ohio State University, Columbus, OH 43210, USA; anthony.kantaras@osumc.edu; 5 School of Psychology, University of Southern Mississippi, Hattiesburg, MS 39406, USA; megan.renna@usm.edu; 6 Department of Psychological Science, University of North Carolina at Charlotte, Charlotte, NC 28213, USA; jbenne70@uncc.edu; 7 Northwell Health, New York, NY 10022, USA; calfano3@northwell.edu; 8 The Ohio State University Comprehensive Cancer Center, The Ohio State University College of Medicine, The Ohio State University, Columbus, OH 43210, USA; stephen.povoski@osumc.edu (S.P.P.); doreen.agnese@osumc.edu (D.M.A.); robert.wesolowski@osumc.edu (R.W.); william.carson@osumc.edu (W.E.C.III); nicole.williams@osumc.edu (N.O.W.); raquel.reinbolt@osumc.edu (R.E.R.); sagar.sardesai@osumc.edu (S.D.S.); anne.noonan@osumc.edu (A.M.N.); daniel.stover@osumc.edu (D.G.S.); mathew.cherian@osumc.edu (M.A.C.); 9 Division of Surgical Oncology, Department of Surgery, The Ohio State University College of Medicine, The Ohio State University, Columbus, OH 43210, USA; 10 Center for Breast Cancer, Yale Cancer Center, Yale University, New Haven, CT 06519, USA; maryam.lustberg@yale.edu; 11 Department of Internal Medicine, The Ohio State University College of Medicine, The Ohio State University, Columbus, OH 43210, USA; 12 Department of Psychiatry and Behavioral Health, The Ohio State University College of Medicine, The Ohio State University, Columbus, OH 43210, USA

**Keywords:** inflammation, depression, LBP, mood, attention, memory, executive function

## Abstract

**Simple Summary:**

Depression and cognitive problems are common in breast cancer survivorship and reduce quality of life. Depression, a risk factor for cognitive decline, is often accompanied by elevated inflammation and a “leaky gut”, which can also impact cognitive function. This study assessed whether depression in tandem with either elevated inflammation or intestinal permeability predicted poorer subjective or objective cognitive function among breast cancer survivors. In secondary analyses of data from 613 survivors with 1015 total study visits, we found that depression combined with either heightened inflammation or intestinal permeability enhanced subjective cognitive complaints, especially focus problems. On neuropsychological tests, depressed survivors performed worse regardless of inflammation or intestinal permeability. These findings suggest that survivors with depression accompanied by immune dysregulation may be more aware of depression-related cognitive deficits compared to other depressed survivors.

**Abstract:**

About one-in-three breast cancer survivors have lingering cognitive complaints and objective cognitive impairment. Chronic inflammation and intestinal permeability (i.e., leaky gut), two risk factors for cognitive decline, can also fuel depression—another vulnerability for cognitive decline. The current study tested whether depression accompanied by high levels of inflammation or intestinal permeability predicted lower subjective and objective cognitive function in breast cancer survivors. We combined data from four breast cancer survivor studies (*n* = 613); some had repeated measurements for a total of 1015 study visits. All participants had a blood draw to obtain baseline measures of lipopolysaccharide binding protein—a measure of intestinal permeability, as well as three inflammatory markers that were incorporated into an inflammatory index: C-reactive protein, interleukin-6, and tumor necrosis factor-α. They reported depressive symptoms on the Center for Epidemiological Studies depression scale (CES-D), and a binary variable indicated clinically significant depressive symptoms (CES-D ≥ 16). The Kohli (749 observations) and the Breast Cancer Prevention Trial (591 observations) scales assessed subjective cognitive function. Objective cognitive function tests included the trail-making test, Hopkins verbal learning test, Conners continuous performance test, n-back test, FAS test, and animal-naming test (239–246 observations). Adjusting for education, age, BMI, cancer treatment type, time since treatment, study visit, and fatigue, women who had clinically elevated depressive symptoms accompanied by heightened inflammation or intestinal permeability reported poorer focus and marginally poorer memory. However, poorer performance across objective cognitive measures was not specific to inflammation-associated depression. Rather, there was some evidence of lower verbal fluency; poorer attention, verbal learning and memory, and working memory; and difficulties with visuospatial search among depressed survivors, regardless of inflammation. By themselves, inflammation and intestinal permeability less consistently predicted subjective or objective cognitive function. Breast cancer survivors with clinically significant depressive symptoms accompanied by either elevated inflammation or intestinal permeability may perceive greater cognitive difficulty, even though depression-related objective cognitive deficits may not be specific to inflammation- or leaky-gut-associated depression.

## 1. Introduction

Troubling cognitive symptoms and objective deficits can persist even years after breast cancer treatment [1,2]. These symptoms are often tied to chemotherapy treatment (i.e., “chemobrain”, “chemo-fog”). However, the magnitude of these chemotherapy-related deficits depends on the type of control group (i.e., healthy control or chemotherapy-free cancer patients) and adjustment for pretreatment performance [3]. Also, a recent systematic review found that cognitive deficits in breast cancer survivorship may not be specific to chemotherapy treatment; in fact, about one-quarter of patients show evidence of cognitive impairment before chemotherapy, and even those treated with other types of therapy may have cognitive declines throughout the disease [2]. Overall, a third of women may have clinically significant cognitive impairment in breast cancer survivorship, and the prevalence is even higher—close to 50%—when considering patient complaints (i.e., subjective cognitive function) [4,5].

Subjective and objective cognitive functioning may provide distinct information, as the two are often unrelated among cancer patients [6,7], with some notable exceptions [8]. Despite these differences in subjective and objective cognitive functioning, subjective cognitive problems are important and meaningful. In fact, Savard and Ganz asserted that patient cognitive complaints should be a primary consideration in research and the clinic, just as psychiatric disorder diagnostic criteria include subjective distress [9]. Moreover, as neurological abnormalities emerge, people may be initially able to compensate and perform normally on objective tests, whereas self-report measures may detect subtle changes that foreshadow decline [10]. Qualitative interviews among survivors one year post-chemotherapy treatment indicated that perceived cognitive impairment (i.e., subjective cognitive function) impaired quality of life [11]. Women primarily expressed concerns about short- and long-term memory, verbal fluency, executive functioning, processing speed, and concentration. These lingering symptoms became especially concerning after other treatment-related symptoms had subsided. Survivors expressed frustration about these perceived impairments, and some indicated that cognitive problems reduced their self-confidence, made them feel as if they were not the same person, negatively affected their relationships, and made work tasks more effortful [11].

Elevated depressive symptoms may, at least transiently, affect one-third of breast cancer survivors [12], and depression may be a risk factor for poorer cognition in survivorship, as it is in the general population [13]. In fact, depression-initiated cognitive impairment may persist even after depression remits [13]. One report suggested that depressive symptoms may be an even stronger predictor of subjective cognitive complaints than cancer treatment type among survivors [14]. Also, our lab previously reported that loneliness, a harbinger of depression [15], predicted poorer subjective memory and concentration and worse performance on a measure of sustained attention among breast cancer survivors [16]. Therefore, the current body of literature points to depression as a potential risk factor for cognitive issues throughout survivorship.

Although cognitive symptoms such as indecisiveness are part of the depression diagnostic criteria [17], there are 227 possible symptom profiles for depression [18], many of which do not include cognitive dysfunction. These different symptom profiles may reflect different etiological pathways. Clinically elevated inflammation accompanies at least 25% of depression cases [19], and the two may reciprocally reinforce one another [20]—a difficult-to-treat immune-mediated depression subtype. Previous studies have demonstrated a relationship between higher inflammation and cognitive deficits among depressed individuals [21,22,23]. Depression and inflammation may each heighten the risk for cognitive problems; thus, the combination may be particularly detrimental. Indeed, a recent cross-sectional study of almost 50,000 adults aged 18 to 93 found that depression and C-reactive protein (CRP, a commonly used inflammatory marker) both independently and jointly predicted poorer performance on the Ruff figural fluency test, a measure of executive function [24]. That is, those who were depressed with higher levels of CRP performed worse than the non-inflamed depressed participants. However, the size of the combined effect was small, especially given the large sample size. Even so, this study provides initial evidence that poorer executive function may accompany the inflammatory subtype of depression.

Chronic inflammation is common among breast cancer survivors. One-third have clinically elevated CRP (>3 mg/L) [25]. About the same percentage may, at least temporarily, struggle with elevated depressive symptoms. Given the high prevalence of depression, inflammation, and cognitive problems among breast cancer survivors, the combined effect of depression and inflammation on subjective and objective cognitive function is perhaps of particular interest in this population. Indeed, a recent longitudinal study with breast cancer survivors and controls showed that higher baseline (pre-treatment) CRP levels predicted poorer subjective cognitive function at annual visits over the next five years only among cancer patients and not controls [26]. Inflammation’s cognitive effect was not more pronounced among depressed survivors. Compared to controls, survivors had poorer objective cognitive function as well, but inflammation did not play as much of a role, except on Trail-Making Test B [26]. In short, current evidence suggests that inflammation may uniquely predict subjective cognitive outcomes among breast cancer survivors.

This study built on the Mac Giollabhui et al. [24] and Carroll et al. [26] studies discussed above. Firstly, it explored the combined effect of depression and inflammation on subjective and objective cognitive function among a large sample of breast cancer survivors assessed at various points throughout survivorship. Unlike these prior studies, which only assessed CRP levels, our study additionally incorporated two other markers of inflammation, interleukin-6 (IL-6), and tumor necrosis factor-α (TNF-α), into an inflammatory index to better capture systemic inflammation. Additionally, our study included a wide variety of cognitive outcomes, including objective and subjective measures. Secondly, as an exploratory aim, our study examined the combined effect of depression and intestinal permeability on subjective and objective cognition. Intestinal permeability (i.e., gut leakiness) occurs when the intestine’s epithelial lining loses its integrity as the tight junctions between cells widen, permitting molecules from the lumen, such as components of bacteria’s outer membrane, to circulate in the bloodstream and migrate to other tissues [27]. Gut leakiness can chronically burden the immune system and may increase the risk of the inflammatory subtype of depression. In fact, we previously reported that female breast cancer survivors and non-cancer controls who had more intestinal permeability reported more depressive symptoms one and two years later, especially if they had elevated levels of inflammation at baseline [28]. Prior research from our lab and others has shown that intestinal permeability may negatively impact cognitive function, both acutely (i.e., after a high-fat meal) [29] and chronically [30]. Therefore, the combination of depression and intestinal permeability may be especially detrimental. Taken together, we hypothesized that depression accompanied by high levels of inflammation or intestinal permeability would predict poorer subjective and objective cognition among breast cancer survivors.

## 2. Materials and Methods

### 2.1. Participants

We conducted secondary analyses of four parent studies (design and aims described below). A total of 613 participants were primarily recruited through the OSU Stefanie Spielman Comprehensive Breast Center, and secondarily recruited through the Army of Women website. Study 1 included 202 women who had Stage 0-IIIC cancer. They completed three visits for a total of 435 study visits between June 2008 and February 2014: one after diagnosis but prior to cancer treatment, and two follow-up visits 6 and 18 months after treatment ended. Compared to the other studies, Study 1 had fewer exclusion criteria than the other studies, yielding a less healthy and more diverse sample (Table 1). Study 2 was the waitlist control group (*n* = 100, Stage 0-IIIA) of a randomized, controlled trial assessing the impact of a 12-week yoga intervention on mood, fatigue, and inflammation among breast cancer survivors [31]. They completed three visits—baseline, three, and six months later—for a total of 266 study visits between October 2007 and December 2012. Study 3 included data from a randomized crossover trial testing predictors of inflammatory responses to the typhoid vaccine [32]. This study featured 162 postmenopausal breast cancer survivors (Stage I-IIIA) who were one to nine years past primary cancer treatment. They completed two visits about one month apart between January 2014 and April 2021. The typhoid vaccine can affect cognition [33], so only data from the placebo visit were used (162 study visits). Study 4 included 149 breast cancer survivors who were diagnosed with Stage I-IIIA cancer. They completed two visits for a total of 252 study visits between June 2014 and April 2021: one after surgery but before adjuvant treatment, and another 2.5 years later. The parent study tested treatment type and depression as predictors of cardiometabolic disease risk [34]. In sum, data from 1015 study visits were used in the current study. The Ohio State University Institutional Review Board approved all studies, and all women gave written informed consent prior to participation.

### 2.2. Procedure

For all studies, participants arrived at the Ohio State University Clinical Research Center between 07:00 and 09:00, and shortly thereafter a nurse completed the blood draw. In Study 1, participants then completed questionnaires relevant to this study (Center for Epidemiological Studies Depression Scale CES-D; RAND 36-item Short Form Survey, SF-36; and the Kohli memory and focus scales) and the parent study. After the blood draw, Study 2′s participants ate a standardized breakfast and reported their depressive symptoms on the CES-D. They then completed other tasks for the parent study, including a 20-min relaxation period and a 20-min speech stressor (only at the baseline visit). Next, they reported their cognitive symptoms on the Revised Breast Cancer Prevention Trial cognitive symptom checklist (BCPT), Kohli, and the SF-36. The remainder of the visit included tasks relevant to the parent study, including a physical flexibility assessment. In Study 3, participants completed a 30-min relaxation period prior to the blood draw. They then received the placebo injection and completed the CES-D, SF-36, and Kohli, followed by a standardized breakfast and a 20-min relaxation period. After this period, they completed other questionnaires for the parent study, and they reported their cognitive symptoms on the BCPT only at Visit 2. For the remainder of the visit, they completed additional tasks for the parent study, including a limited battery of neuropsychological tests. These tests were completed five to seven hours after the baseline blood draw, and inflammation significantly increased throughout the day for both the vaccine and placebo [32]. Therefore, we did not include these neuropsychological tests in the current study. In Study 4, women completed an exercise stress test a few days prior to each study visit for the purposes of the parent study. At these visits, they reported their fatigue on the SF-36. For their main study visits, participants completed a 30-min relaxation period prior to the blood draw. They then completed the objective neuropsychological testing battery, ate a standardized breakfast, completed other tasks relevant to the parent study, and reported their depressive symptoms on the CES-D and cognitive problems on the BCPT.

### 2.3. Measures

#### 2.3.1. Subjective Tests

The Kohli scale assesses concentration and memory in cancer patients, with higher scores indicating worse cognitive function [10]. Patients rated their memory and concentration problems over the past five days on separate 11-point Likert scale ranging from 0 (“not present”) to 10 (“as bad as you can imagine”), with a score of 7 or greater being categorized as “severe” [10].

The Revised Breast Cancer Prevention Trial (BCPT) cognitive function subscale is a reliable evaluation of perceived impairment in several cognitive domains, including memory, concentration, and attention, among breast cancer populations [35]. Patients reported how bothered by cognitive symptoms (i.e., forgetfulness, difficulty concentrating, distractibility) they were in the past four weeks on a 5-point Likert scale from 0 (not at all) to 4 (extremely). A higher score on the composite score across the three domains indicates greater perceived impairment [35].

#### 2.3.2. Objective Tests

Trail-Making Tests (TMT) A and B are highly sensitive measures used to detect cognitive impairment [36]. The TMT assesses complex visual scanning with a motor component heavily reliant on motor speed and agility [37]. The test requires the participant to connect numbered (A) and numbered and lettered (B) circles in ascending order. The time taken to complete the test is an indicator of cognitive function, with longer times indicating poorer cognitive function [36].

The FAS test and the animal-naming test measure verbal fluency. The FAS test requires the participant to list as many words as possible that begin with the letters F, A, and S in one minute, excluding proper nouns, numbers, and words stemming from the same root [38]. Similarly, the animal-naming test requires the participant to list as many animals as possible in one minute, excluding repetitions [39].

The Conners continuous performance test third edition (CPT-3) is a computer-based test of sustained attention [40]. CPT-3 requires the participant to press a button when a specific target stimulus “X” is presented and to inhibit their response when any other letter is presented. The 15-min test includes 360 trials designed to measure the ability to distinguish target from non-target stimuli (i.e., detectability), the number of false alarms (i.e., commission errors), and the number of missed targets (i.e., omission errors). Response times are recorded, with particular emphasis on the response times for target stimuli (i.e., hit reaction time). For all variables, T-scores between 45 and 54 are average. For hit reaction time, scores below 45 are fast and scores above 54 are slow; for all other variables, T-scores of 60 and above are elevated [41]. The CPT-3 is one of the most widely used assessments for continuous performance and has a strong internal consistency [41,42].

The revised Hopkins verbal learning test (HVLT) is a brief assessment of verbal learning and memory [43]. The HVLT assesses mild cognitive impairments across numerous populations, including the elderly, those with traumatic brain injury, and cancer patients [44]. The test consists of 12 nouns—with 4 words each from one of three semantic categories—presented in three learning trials followed by a delayed recall trial and a final recognition trial completed 20 min after the final learning trial. In the recognition trial, the participant is asked to recognize words from the original list on a new list of 24 words that includes the 12 original nouns, as well as 12 that were not on the original list (6 semantically related, 6 semantically unrelated). The primary outcome measures from the HVLT include total recall (the sum of words recalled from the first three learning trials), percent retained (number of words recalled on the delayed-recall trial divided by the greatest number recalled on the second or third learning trial; quotient is multiplied by 100), and recognition (true positives minus false positives).

The n-back test measures working memory [45]. The task presents a sequence of letters and requires participants to indicate whether each stimulus matches the one presented *n* (one or two) trials back. A lower accuracy indicates a poorer working memory, while slower response times indicate a higher cognitive load or difficulty with the task [46].

#### 2.3.3. Depression

The 20-item Center for Epidemiological Studies-Depression (CES-D) scale assesses the frequency of depressive symptoms (e.g., decreased appetite, restless sleep) using a Likert scale from 0 (rarely or none of the time) to 3 (most or all of the time). Scores range from 0 to 60 with the greater score correlating with a higher prevalence of depressive symptoms [47]. A clinical cutoff score of 16 indicates clinically significant depressive symptoms [48]. Throughout the manuscript, we refer to those above the clinical cut-score as “depressed,” but it is important to note that they may not have met diagnostic criteria for clinical depression. Given our hypotheses, it is important to note that the CES-D has only one item that involves cognition (i.e., “I had trouble keeping my mind on what I was doing”).

#### 2.3.4. Biological Markers

To control for diurnal variation, fasting blood samples were collected between 07:00 and 09:00. Assay information, including sensitivities and inter- and intra-assay coefficients of variation (CoV), are included in Table 2. Note that the samples from Studies 1 and 2 were run together, as were the samples from Studies 3 and 4. All CoVs fell below 10%, a common benchmark [49], except for LBP in Studies 3 and 4, which was just above 10%. A chemiluminescence method via the Immulite 1000 (Siemens Healthcare Diagnostics, Inc., Deerfield, IL, USA) quantified serum levels of C-reactive protein (CRP). Tumor-necrosis factor-α (TNF-α), interleukin-6 (IL-6), and lipopolysaccharide-binding protein (LBP) were measured with Meso Scale Discovery Kits using an electrochemiluminescence method; they were read via the Meso Scale Discovery Sector Imager 2400 (Meso Scale Discovery, Rockville, MD, USA). CRP, TNF-α, and IL-6 are some of the most common inflammatory markers reported in the literature; they are also related, as the latter two induce the liver to produce CRP. We measured LBP as an index of intestinal permeability, as we have performed previously [28], because most lipopolysaccharide (LPS) resides in the gastrointestinal tract, as it is a shell component of Gram-negative bacteria [50]; therefore, serum levels may indicate a poorer gut barrier integrity (i.e., leaky gut) [51]. LPS has a short half-life and is difficult to measure in humans [52]; thus, LBP, which is a more stable marker, was used as a proxy. LBP binds LPS and presents it to CD-14 receptors on monocytes and macrophages, which facilitates proinflammatory signaling. LBP is associated with LPS and other intestinal permeability markers [53,54,55], and it is a clinical marker of endotoxemia that provides information about the risk for accelerated aging, metabolic syndrome, and gastrointestinal disorders [52,56].

#### 2.3.5. Covariates

At each study visit, a nurse measured participants’ height and weight. Medical records provided cancer treatment information, including treatment type (surgery only, surgery and chemotherapy, surgery and radiation, surgery plus chemotherapy and radiation) and months since treatment. Participants reported their highest education level (high school or less, some college, college graduate, graduate or professional training) at the initial visit. At each visit, they completed the widely-used RAND 36-Item Health Survey (SF-36) [57], which includes the four-item energy/fatigue subscale. Scores range from 0 to 100, with higher scores indicating more energy. Both depression and inflammation can promote lower energy; thus, we adjusted for self-reported fatigue to ensure that any observed associations with cognition were not simply due to fatigue.

### 2.4. Analytic Method

Supplemental Table S1 shows pairwise correlations between the study variables of interest. Due to the clustering of observations by subject, we used bootstrapping to obtain the significance. We also used chi-square and ANOVA tests to compare the four samples on demographic variables of interest at Visit 1.

In models involving the inflammatory index as a predictor, 427 women (749 visits from Study 1, 2, and 3) were included when modeling Kohli outcomes; 322 women (591 visits from Study 2, 3, and 4) were included when modeling BCPT cognitive function; and between 145 and 148 participants (239–246 visits from Study 4) were included when modeling neuropsychological testing outcomes. In models involving LBP as a predictor, 419 participants (730 visits from Study 1, 2, and 3) were included when modeling Kohli outcomes; 303 participants (566 visits from Study 2, 3, and 4) were included when modeling BCPT cognitive function; and between 137 and 140 (228–239 visits from Study 4) were included when modeling neuropsychological testing outcomes due to some LBP missingness. Table 3 shows the available data by study visit. Compared to those who did not have data to be included in at least one model (*n* = 16), the included participants (*n* = 613) received different cancer treatment regimens (*p* = 0.03) and were less likely to take antidepressants (*p* = 0.04); excluded participants did not differ from included participants on any other variables of interest (*p*s > 0.06).

To minimize statistical tests, inflammation was quantified via an inflammatory index, which was created by z-scoring the natural log-transformed values of CRP, TNF-α, and IL-6, and then calculating the average of these z-scores, as described in other work [58]. In our sample, IL-6 was weakly to moderately correlated with the other inflammatory markers (0.17 < *r*s < 0.47) (Supplemental Table S1). However, TNF-α was not correlated with CRP in this sample. Even so, the inflammatory index provides a more global estimation of inflammatory status than any one marker alone. Although some prior research has excluded or winsorized CRP values greater than 10 mg/L due to concerns that these values indicated acute illness, recent evidence suggests that acute illness may not always cause such elevations [59]; therefore, we included the entire range of inflammatory markers, but we log-transformed them (base e) such that substantially elevated values would not have an undue influence on the results. The inflammatory index variable was calculated for all participants that had all three inflammatory variables across all relevant study visits: all visits from Study 1, Study 2′s waitlist control group study visits, Study 3′s placebo visit, and both visits from Study 4. Past relevant work used CRP alone [24,26]; thus, we conducted sensitivity tests using only natural log-transformed CRP as the predictor (in an interaction with depression). In Section 3, we report cases in which these results differ. Also, we dichotomized the CES-D scores based on the established cutoff score of 16 [48] in line with prior relevant work that used the current depression status rather than the continuous symptoms [24].

To investigate the questions of interest, we used generalized estimating equations (GEE) with an identity link and robust standard errors [60,61]. GEE models can handle the discrete nature of some of our primary outcomes and produce efficient, unbiased estimates of how much the average response changes per one-unit increase in the predictor [60,61]. GEE models also account for within-subject correlations arising from repeated measurements. The predictors of interest were the interactions between clinically significant depressive symptoms (binary CES-D cutoff score) with inflammation and, in separate models, with LBP (i.e., gut leakiness). Outcomes of interest were the subjective and objective cognitive variables, in separate models. Importantly, our models were cross-sectional, in that they included data from the repeated study visits, but depression, inflammation, and gut leakiness from one visit were used to model cognitive outcomes at the same visit. We probed significant interactions at ±1 standard deviation from the mean of inflammation and gut leakiness for those who were depressed and not depressed. Below, we also report significant main effects for inflammation and depression, taken from the models that include their interaction term, as well as the main effect for LBP, taken from the model that includes its interaction with depression. Contrasts within the models containing the interaction were used to obtain these estimated main effects, resulting in effects that average over the other predictor (e.g., the main effect for depression is at the mean for inflammation; the mean effect for inflammation is the averages for those above below the binary CES-D cutoff score estimates). All primary models were adjusted for education, cancer treatment type, months since treatment, fatigue, BMI, age, and visit within the study. Due to complex inter-relationships among BMI, fatigue, and our variables of interest, we also conducted sensitivity analyses to see whether excluding these two covariates changed the pattern of results. The results were largely unchanged (Supplemental Table S2). Alpha levels were set at 0.05.

## 3. Results

### 3.1. Demographic Information

From the entire sample, 8% (*n* = 46) had Stage 0, 46% (*n* = 283) had Stage 1, 39% (*n* = 240) had Stage II, and 7% (*n* = 43) had Stage III breast cancer. The breakdown of cancer treatment type was as follows: 23% (*n* = 139) had surgery only, 19%, (*n* = 115) received surgery and chemotherapy, 27%, (*n* = 163) had surgery and radiation, and 32% (*n* = 196) had all three. Women completed study visits between 32.9 months prior to and 131.3 months after treatment, with a mean of 11.6 months post-treatment (SD = 25.9). Survivors’ ages ranged from 26 to 88 years (*M* = 54.4, *SD* = 10.2), and the average participant was overweight (BMI *M* = 28.3, *SD* = 6.4). Most participants were White (86%, *n* = 525), followed by Black (10%, *n* = 63), Asian (2%, *n* = 13), Native American (1%, *n* = 5), Mixed (1%, *n* = 6), and Other (<1%, *n* = 1). A majority of the sample was highly educated: 62% graduated from college, including 31% who pursued graduate or professional training. In total, 8.9% and 10.5% of participants reported severe focus and memory problems, respectively, on the Kohli scale during at least one visit. Across all study visits, CES-D scores had a large variability (0–49) with a mean of 11.1 (*SD* = 9.1). Using the cut-off score of 16, 26% (*n* = 285) met the criteria for clinically significant depressive symptoms. In terms of inflammation, the mean CRP value was above the clinical cutoff point of 3 mg/L (*M* = 3.4 mg/L, *SD* = 8.2). Out of the 1152 CRP observations across studies, 27 of them were above 20 mg/L, 4 were above 50 mg/L, and 1 was above 100 mg/L. Elevated inflammation (CRP > 3 mg/L) was more common at visits in which participants were depressed (χ^2^(1) = 5.29, *p* = 0.022): At 34.0% of visits in which participants were depressed, they also had clinically elevated CRP levels, whereas they had elevated CRP at only 25.8% of visits in which they were not depressed. Table 4 includes demographic information, Table 5 shows average depression and inflammation levels across the full sample, and Table 6 displays the cognitive outcome values across the full sample.

### 3.2. Subjective Tests

#### 3.2.1. Depression and Inflammation

**Figure 1:**
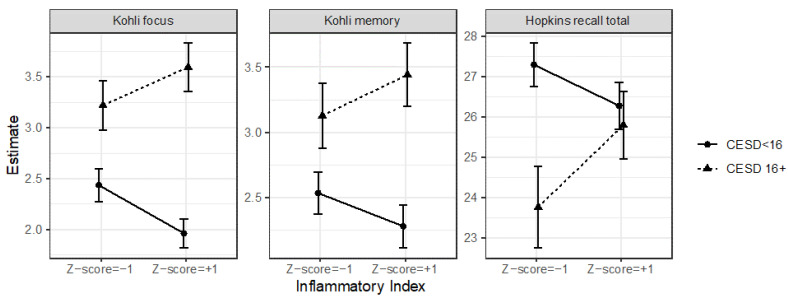
Depression and inflammation interacted to predict cognitive function. The depressed women with the highest levels of inflammation reported the poorest cognitive functioning on the Kohli focus scale and Kohli memory scale. The combination of depression and heightened inflammation also predicted Hopkins total recall, but in the opposite direction as expected, such that depressed women recalled fewer words than non-depressed women only if they had low and average, but not high, levels of inflammation.

**Figure 2:**
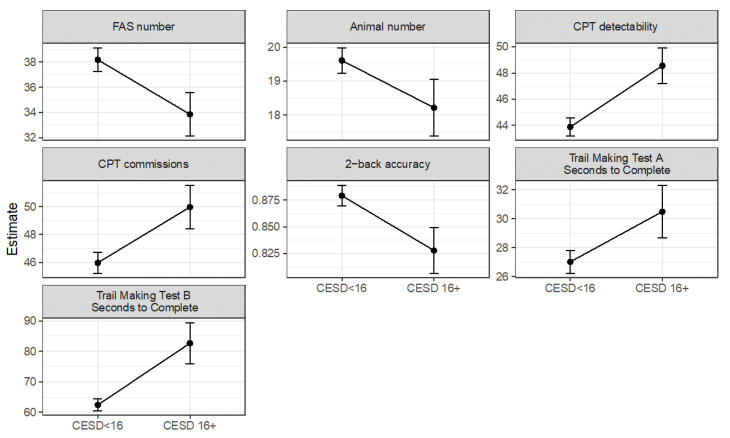
Depressed breast cancer survivors had worse objective cognitive function. Across a wide variety of neurocognitive tests depicted below, depressed survivors performed worse than non-depressed survivors. CPT = Conners continuous performance test, CESD = Center for Epidemiological Studies Depression Scale.

Depression’s relationship with the Kohli focus scores depended on the level of inflammation (χ^2^(1) = 6.65, *p* = 0.010). There was also a similar but non-significant trend for Kohli memory scores (χ^2^(1) = 2.85, *p* = 0.091), but not BCPT (*p* = 0.22). Although depression predicted memory and focus problems at all levels of inflammation (*p*s < 0.021), the effects were strongest for those with high inflammation. See Figure 1. In these same models, those who were depressed reported poorer cognitive function on the BCPT (*B* = 0.43, *SE* = 0.11, χ^2^(1) = 17.54, *p* ≤ 0.001), Kohli focus (*B* = 1.21, *SE* = 0.20, χ^2^(1) = 36.14, *p <* 0.001), and Kohli memory scales (*B* = 0.88, *SE* = 0.20, χ^2^(1) = 19.08, *p <* 0.001). See Figure 2. However, inflammation alone did not predict subjective cognitive function (*p*s > 0.79). In sensitivity analyses, when substituting CRP for the inflammatory index, the results were less compelling: There was only a marginal interaction between CRP and depression to predict Kohli focus (*p* = 0.095), and the combination of CRP and depression did not predict Kohli memory (*p* = 0.56); otherwise, the results were not different.

#### 3.2.2. Depression and Intestinal Permeability

**Figure 3:**
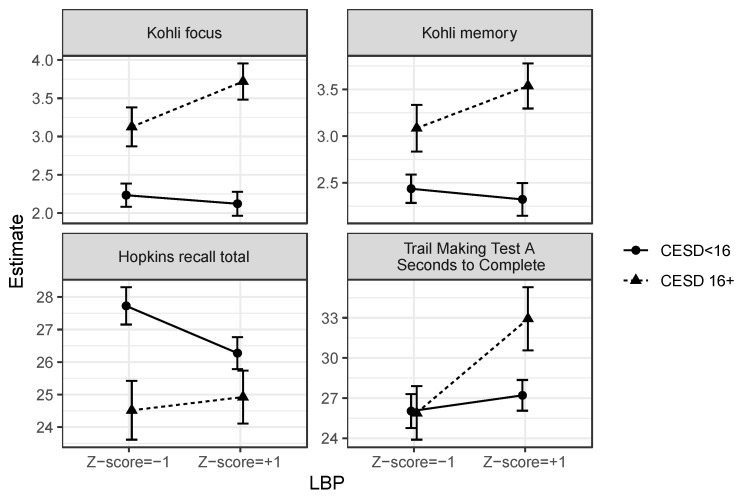
Depression interacted with lipopolysaccharide-binding protein (LBP) to predict cognitive function. Depressed women with greater intestinal permeability reported poorer focus and memory and were slower to complete Trail Making Test A. The combination of depression and heightened intestinal permeability also predicted Hopkins total recall, but in the opposite direction as expected, such that depressed women recalled fewer words than non-depressed women only if they had low and average, but not high, levels of LBP.

The results were similar for the combination of depression and intestinal permeability. There was a significant interaction between depression and intestinal permeability to predict focus problems (χ^2^(1) = 4.32, *p* = 0.038) and to marginally predict memory problems (χ^2^(1) = 2.95, *p* = 0.086), but not BCPT scores (*p* = 0.93). Specifically, the relationship between depression and focus and memory problems was stronger for women with more intestinal permeability. See Figure 3. In these models, LBP did not have a direct relationship with subjective cognitive function (*p*s > 0.12).

### 3.3. Objective Tests

#### 3.3.1. Depression and Inflammation

The combination of heightened inflammation and depression did not predict objective cognitive performance on the animal-naming test (*p* = 0.80), FAS test (*p* = 0.45), CPT commission errors (*p* = 0.59), CPT omission errors (*p* = 0.40), CPT detectability (*p* = 0.91), CPT hit reaction time (*p* = 0.35), 1-back accuracy (*p* = 0.52), 2-back accuracy (*p* = 0.56), 1-back response time (*p* = 0.30), 2-back response time (*p* = 0.44), HVLT recognition (*p* = 0.38), HVLT percent retained (*p* = 0.66), or Trail A (*p* = 0.85) or B (*p* = 0.47) response times. The combination of high inflammation and depression predicted HVLT total recall (χ^2^(1) = 5.15, *p* = 0.023), such that those who were depressed recalled fewer words only if they had average (*B* = −2.00, *SE* = 0.67, χ^2^(1) = 8.90, *p* = 0.003) and low (*B* = −3.53, *SE* = 1.02, χ^2^(1) = 11.91, *p* = 0.0006), but not high (*p* = 0.58), levels of inflammation (Figure 1).

Those who were depressed had more CPT commission errors (*B* = 4.01, *SE* = 1.54, χ^2^(1) = 6.82, *p* = 0.009), were less able to detect target from non-target stimuli on the CPT (*B* = 4.66, *SE* = 1.40, χ^2^(1) = 11.14, *p* = 0.0008), were less accurate on the 2-back (*B* = −0.05, *SE* = 0.02, χ2(1) = 4.88, *p* = 0.027), named fewer words on the FAS test (*B = −*4.33, *SE* = 1.71, χ^2^(1) = 6.43, p = 0.011), named marginally fewer animals on the animal-naming test (*B = −*1.40, *SE* = 0.85, χ^2^(1) = 2.72, *p* = 0.099), recalled fewer words on the HVLT (*B* = −2.00, *SE* = 0.67, χ^2^(1) = 8.90, *p* = 0.003), were slower on the Trail B test (*B* = 20.27, *SE* = 7.05, χ^2^(1) = 8.26, *p* = 0.004), and were marginally slower on the Trail A test (*B* = 3.47, *SE* = 1.87, χ^2^(1) = 3.44, *p* = 0.064) than those who were below the depressive symptom clinical cutoff score (Figure 2). Neither depression (*p*s > 0.11) nor inflammation (*p*s > 0.17) independently predicted performance on any other neuropsychological test, except that those who were more inflamed named marginally fewer animals (*B* = −0.88, *SE* = 0.52, χ^2^(1) = 2.91, *p* = 0.088) and were marginally less accurate on the 2-back (*B* = −0.03, *SE* = 0.014, χ^2^(1) = 3.30, *p* = 0.069).

In sensitivity analyses, substituting CRP for the inflammatory index did not change the pattern of results for the interaction term. In terms of main effects, CRP, unlike the inflammatory index, did not marginally predict animal naming (*p* = 0.11) or 2-back accuracy (*p* = 0.25), but the other results were similar.

#### 3.3.2. Depression and Intestinal Permeability

The combination of depression and intestinal permeability did not track with objective cognitive performance on the animal-naming test (*p* = 0.85), FAS test (*p* = 0.28), CPT commission errors (*p* = 0.69), CPT omission errors (*p* = 0.76), CPT detectability (*p* = 0.79), CPT hit reaction time (*p* = 0.36), 1-back accuracy (*p* = 0.90), 2-back accuracy (*p* = 0.74), 1-back response time (*p* = 0.54), 2-back response time (*p* = 0.18), HVLT recognition (*p* = 0.33), HVLT percent retained (*p* = 0.93), or Trail B completion time (*p* = 0.23). There were two marginal effects: Depression and intestinal permeability interacted to marginally predict the Trail A completion time (χ^2^(1) = 3.56, *p* = 0.059) and HVLT total recall (χ^2^(1) = 3.00, *p* = 0.084). That is, depression predicted a slower Trail A completion time only at higher levels of LBP (*B* = 5.72, *SE* = 2.22, χ^2^(1) = 36.64, *p* = 0.010), but not at average (*p* = 0.12) or low (*p* = 0.96) LBP. In contrast, depression predicted a poorer HVLT recall only at average (*B* = −2.28, *SE* = 0.72, χ^2^(1) = 9.92, *p* = 0.002) and low (*B* = −3.21, *SE* = 0.96, χ^2^(1) = 11.18, *p* = 0.0008), but not high (*p* = 0.11) LBP. Also, in the same models, those with greater intestinal permeability had a slower response time on the 2-back (*B* = 22.11, *SE* = 9.14, χ^2^(1) = 5.85, *p* = 0.016), slower Trail A completion time (*B* = 2.05, *SE* = 0.91, χ^2^(1) = 5.07, *p* = 0.024), marginally lower HVLT percent retained (*B* = −2.98, *SE* = 1.63, χ^2^(1) = 3.36, *p* = 0.067), and marginally poorer HVLT recognition (*B* = −0.11, *SE* = 0.07, χ^2^(1) = 2.85, *p* = 0.092). See Figure 3. Intestinal permeability did not have a direct relationship with any other objective outcome (*p*s > 0.10). See Table 7 for a results summary.

## 4. Discussion

In this study among breast cancer survivors at many different stages of survivorship, we found that those with the combination of depression and heightened inflammation or intestinal permeability (i.e., gut leakiness) reported more subjective focus problems and marginally more memory problems. In these same models, depression also emerged as a significant independent predictor of subjective cognitive problems. However, among the subsample that had neuropsychological testing data, only depression alone—not in concert with inflammation or gut leakiness—emerged as a reliable predictor of performance. Survivors who were more depressed had poorer attention on the CPT, lower verbal fluency on the FAS test and animal-naming test, worse recall on the HVLT, poorer working memory on the 2-back, and more difficulties with visuospatial search on the trail-making tests. In contrast to other literature [21,22,23,26], inflammation alone did not reliably predict neuropsychological testing performance, except for two marginally non-significant effects. These null results are surprising considering our inclusion of three inflammatory variables to better index the inflammatory burden, compared to other studies that have only used a single marker. In contrast to inflammation, LBP, our marker of intestinal permeability, predicted a few objective outcomes: Greater LBP was associated with slower responses on the 2-back, slower Trail A completion times, and a marginally poorer verbal memory on the HVLT. Notably, all of our primary results were independent of cancer treatment type, months since treatment, and other demographic and health covariates. In sum, this study showed that (1) elevated depressive symptoms may reliably predict poorer subjective cognitive function and worse neuropsychological testing performance throughout breast cancer survivorship; (2) depressed individuals who also have heightened inflammation or intestinal permeability may be especially burdened by lower perceived cognitive functioning; (3) intestinal permeability may independently predict certain aspects of objective cognitive function (i.e., working memory, visuospatial search); and (4) heightened inflammation or intestinal permeability may not exacerbate depression-related objective cognitive deficits.

These results add to the literature among non-cancer populations, which shows a connection between depression and poorer cognition. Meta-analytic evidence shows that those who are depressed have poorer attention, executive function, and memory than healthy controls—and this difference persists even when depression remits [18]. Here, we showed that breast cancer survivors with clinically elevated depressive symptoms had a poorer verbal fluency, lower verbal (short-term) recall on the HVLT, worse working memory on the n-back, poorer visuospatial search on the trail-making test, and worse attention on the CPT. Additionally, in line with prior work [14], we found that depressed survivors reported poorer focus, memory, and overall cognition than non-depressed survivors. Thus, in our study, depression was a risk factor for poorer objective and subjective cognition across a variety of measures and domains.

Similar to a prior study among the general population [24], but unlike a recent study among breast cancer survivors [26], we found unique effects for the combination of depression and heightened levels of inflammation. That is, depressed survivors with heightened inflammation (perhaps indicative of inflammation-driven depression), reported more focus problems and marginally more memory problems. This result parallels earlier findings among cancer patients undergoing pro-inflammatory cytokine treatment, in which 30% reported moderate to severe concentration issues, and cognitive problems were more pronounced among those with depression; intriguingly, these self-reported cognitive issues were responsive to paroxetine pre-treatment (versus placebo) [62]. Our findings add to this literature, revealing that endogenous inflammation combined with depression also increases the risk of poorer self-reported cognitive function, and especially concentration difficulties. That said, survivors with depression and elevated inflammation did not report being more troubled by cognitive problems on the BCPT. The BCPT models included fewer data points and a slightly different subsample, which may have influenced the results. Furthermore, it assessed cognitive issues over the past month, compared to the last five days on the Kohli scale. Depressive symptoms and inflammation were measured concurrently, so our pattern of results may indicate that the combination of depression and elevated inflammation predicts only concurrent or recent subjective cognitive issues.

Also, as stated above, depression’s relationship with objective outcomes (i.e., neuropsychological testing) did not depend on the levels of inflammation, except for HVLT recall. Depressed survivors had a lower verbal recall only if they had average or low, but not high, levels of inflammation. This finding is contrary to our hypothesis; one possibility is that women with inflammation-associated depression were aware of their impairment, and therefore they expended more effort to compensate. Indeed, in another study, even with mild to moderate levels of cortical amyloid-β deposition, women were able to compensate and perform better on verbal memory tasks than men with the same level of deposition [63].

Our findings for subjective cognition are clinically meaningful: Subjective cognitive impairment may be even more important than neuropsychological test results [9]. There is some evidence that subjective cognitive impairment predicts future objective decline particularly well among women [64]. Moreover, compared to neuropsychological testing, self-report measures may capture more subtle impairment. This point is especially relevant to cross-sectional research, or any instance in which there is no control for baseline (e.g., pre-cancer) neuropsychological test performance; in these cases, survivors’ test results may be within the normal range, yet they may report cognitive difficulties because certain cognitive tasks require more effort. In rating their cognition, survivors may attempt to use their prior (pre-cancer) perceived cognitive functioning as an anchor to rate their current functioning. Thus, in a sense, self-reported outcomes may reflect some aspect of cognitive change over time, with the individual serving as her own control and assessor across time—even in cross-sectional research at a single time point. To our knowledge, this is some of the first evidence among breast cancer survivors that inflammation-associated depression may relate to poorer subjective cognition.

We also report here for the first time that those with clinically elevated depressive symptoms and intestinal permeability report poorer focus and marginally poorer memory. Again, these effects largely did not extend to the neuropsychological testing realm, except for two marginally non-significant effects: Depressed survivors with greater intestinal permeability were slower to complete the Trail A test, indicating more difficulty with visuospatial search; this effect was not evident among depressed survivors with less intestinal permeability. Also, the other marginally non-significant effect was similar to the combined effect of depression and inflammation on HVLT recall: depressed survivors with a lower intestinal permeability had a worse recall—contrary to hypotheses. Even so, these effects on objective cognitive testing were marginally non-significant and should not be overinterpreted. Overall, our pattern of results suggests that those with inflammation- or leaky-gut-related depression may be particularly cognizant of their depression-related cognitive impairment, even though their objective impairment is similar to those with other subtypes of depression.

Unlike prior research [21,22,23,26], our inflammatory index was not directly related to subjective or objective measures. We looked cross-sectionally, whereas inflammation’s cognitive effect may play out longitudinally [26]. Also, many prior studies have focused on CRP, whereas we computed an inflammatory index that takes a more comprehensive account of inflammatory status. In fact, sensitivity analyses substituting CRP for the inflammatory index yielded fewer associations. In terms of intestinal permeability’s direct effect, we found that those with a greater intestinal permeability had slower response times on the 2-back and a slower Trail A completion time, indicating a poorer working memory and visuospatial search, respectively. Interestingly, both outcomes involve response times; therefore, future research should continue to examine whether LBP specifically relates to psychomotor slowing. Of note, we statistically adjusted for levels of fatigue, so this result suggests that greater intestinal permeability predicts slower response times independent of fatigue. Although studies among animals or other clinical populations have reported direct, and even longitudinal, relationships between elevated gut leakiness and poorer cognition [30,65,66,67,68,69], this is one of the first studies to do so among breast cancer survivors.

Past research may help to explain why breast cancer survivors with elevated inflammation or intestinal permeability report more cognitive issues, even though their neuropsychological testing performance is comparable to other depressed survivors. Firstly, inflammation and intestinal permeability may facilitate or at least indicate risk for vascular pathology [70,71], which is already elevated among breast cancer survivors [72], ultimately leading to steeper cognitive decline and even vascular dementia. Secondly, peripheral inflammatory markers, especially CRP, are highly correlated with central inflammatory markers in depressed people; for example, plasma CRP, IL-6, and TNF-α were moderately to highly correlated with CRP in the cerebral spinal fluid (0.35 < *rs* < 0.86) [73]. Central inflammation can reduce synaptic plasticity and impair neurogenesis—the underpinnings of cognitive impairment [74]. Although this impairment may not yet manifest on objective testing, survivors may begin to surmise that cognitive tasks are more difficult—a result of inflammation-related neurobiological changes. Thirdly, peripheral inflammation, intestinal permeability, and depression are all associated with altered blood–brain barrier (BBB) permeability. There is some recent controversy about whether the BBB is more or less permeable in inflammation-associated depression [75]. Reduced permeability implies a lower solute exchange, which could change the brain’s metabolism such that cognitive tasks are perceived as more difficult [75]. Conversely, increased permeability renders the brain more vulnerable to inflammation’s harmful effects. Overall, our findings call for more clinical research investigating the mechanisms underlying subjective cognitive impairment in inflammation- and leaky-gut-associated depression in survivors.

## 5. Strengths: Limitations, and Future Directions

Our significant results are notable because they emerged even after controlling for several relevant covariates, including education, cancer treatment, months since treatment, fatigue, and BMI. We also included survivors at all points in survivorship—from pre-adjuvant treatment to over a decade post-treatment. Thus, depression and its combined effect with inflammation and intestinal permeability predicted poorer cognition throughout the survivorship continuum. Also, inflammation, intestinal permeability, and depression are common among those who are obese, so statistically adjusting for BMI ensured that our effects were the result of inflammation and not simply due to obesity. Other strengths include: the large sample size with over 1000 observations, our full battery of subjective and objective measures, and our use of three rather than one inflammatory marker to index inflammation.

Although our large and heterogenous sample of breast cancer survivors at different points in survivorship is a strength in terms of replicability and generalizability, it is also a limitation in terms of potential confounds and between-sample differences, which cannot be fully eliminated even with careful covariate selection. In terms of other limitations, our analyses were cross-sectional rather than longitudinal because we were concerned that attrition (especially of the sickest) might bias longitudinal results. Also, instead of an interview-assessed psychiatric diagnosis, we used the CES-D cutoff score to index clinically significant depressive symptoms; although widely used as a cutoff, those labeled “depressed” may not have met criteria for a depressive disorder. Another limitation is that only Study 4 had a full battery of neuropsychological testing, so the objective measures were completed among a smaller sample than the subjective measures, which lowered our statistical power to find an effect. The Mac Giollabhui et al. study [24] revealed that the combination of inflammation and depression had only a small effect on objective executive function, so we may not have had enough statistical power to uncover the combined effect in our smaller sample. Even so, we had a more comprehensive testing battery than this prior study. Also, our subjective cognitive measures were short, and these relationships should be tested with longer assessments of subjective cognitive functioning. Lastly, this sample was female, primarily White, and highly educated, so these associations should be explored among more diverse samples. Women may be more susceptible to inflammation’s mood and behavioral effects [76], and they may also have more cognitive impairment when depressed compared to men [77]. Therefore, our findings may not be replicated among a male sample.

## 6. Clinical Implications

Overall, depressed breast cancer survivors may be at risk for both subjective and objective cognitive issues in survivorship. Also, depressed survivors with elevated inflammation or intestinal permeability may be more sensitive to these deficits, leading to more subjective complaints. Subjective cognition is clinically meaningful and may impact self-confidence, social relationships, and overall quality of life; thus, our results suggest that those with inflammation- or leaky-gut-associated depression may need additional support and proactive intervention to address perceived decline. It may be as simple as inquiring about cognitive function and validating concerns: One qualitative study noted that healthcare providers’ and family members’ acknowledgment and validation of survivors’ cognitive complaints may have facilitated adjustment [8]. These results show the importance of identifying and treating depression with the goal of attenuating cognitive declines in survivorship. Our findings also point to the possibility that anti-inflammatory interventions, especially among survivors with elevated levels of inflammation, may help to reduce cognitive complaints, and perhaps increase quality of life, among survivors—an important area for future work. For example, we found that one widely-available over-the-counter dietary supplement, omega-3 fatty acid, not only reduced basal inflammation [78] but also inflammatory responsivity to an acute stressor [79]. Moreover, omega-3 may help to fortify the gut barrier and blood–brain barrier [80,81]—promising areas for future investigation as potential mechanisms to reduce cognitive impairment in survivorship.

## 7. Conclusions

Depression may be a risk factor for both subjective and objective cognitive impairment throughout breast cancer survivorship, and depressed survivors who have elevated inflammation or intestinal permeability may be especially sensitive to these impairments, especially related to concentration. Their subjective complaints are worthy of additional attention in the clinic and lab, as they can greatly reduce quality of life.

## Figures and Tables

**Table 1:** Exclusionary criteria across studies.

	Study 1	Study 2	Study 3	Study 4
Any other cancer besides basal or squamous cell carcinomas	X	X	X	X
Significant visual, auditory, or cognitive impairment that would limit study participation	X	X	X	X
Inflammatory breast cancer		X		
Diabetes		X	X	X
Stroke				X
Current heart disease			X	X
Uncontrolled hypertension		X	X	X
Prior heart attack				X
Heart failure				X
Age (<21 or >75)				X
Heart transplant				X
Other major cardiovascular surgery				X
Liver disease			X	X
Autoimmune and/or inflammatory disease		X	X	X
Alcohol/drug abuse		X	X	X
Steroid use				X
Recent (<3 month) initiation of antidepressant medication				X
Other medical conditions that would limit study participation				X
Distance from lab (>100 miles)				X
Neoadjuvant chemotherapy or radiation treatment				X
Peripheral vascular disease				X
Liver or kidney failure		X		
Symptomatic ischemic heart disease		X		
Anemia		X	X	
Chronic obstructive pulmonary disease		X		
Current yoga practice (within past 6 months)		X		
Previous yoga practice for >3 months		X		
Five or more hours of vigorous physical activity per week		X		
A prior typhoid vaccination			X	
Smoking			X	
Steroids, statins, or other anti-inflammatory meds			X	

**Table 2:** Assay information.

		Study 1	Study 2	Study 3	Study 4
CRP	Intra-assay CoV	3.10%	3.10%	7.23%	7.23%
	Inter-assay CoV	7.30%	7.30%	2.87%	2.87%
	Sensitivity	0.30 mg/L	0.30 mg/L	27.60 pg/mL	27.60 pg/mL
IL-6	Intra-assay CoV	1.43%	1.43%	4.10%	4.10%
	Inter-assay CoV	4.42%	4.42%	6.50%	6.50%
	Sensitivity	0.37 pg/mL	0.37 pg/mL	0.03 pg/mL	0.03 pg/mL
TNF-α	Intra-assay CoV	4.32%	4.32%	8.85%	8.85%
	Inter-assay CoV	5.30%	5.30%	3.30%	3.30%
	Sensitivity	0.26 pg/mL	0.26 pg/mL	0.04 pg/mL	0.04 pg/mL
LBP	Intra-assay CoV	2.74%	2.74%	10.80%	10.80%
	Inter-assay CoV	8.33%	8.33%	4.25%	4.25%
	Sensitivity	0.04 ng/mL	0.04 ng/mL	0.04 ng/mL	0.04 ng/mL

CRP = C-reactive protein, IL-6 = interleukin-6, TNF-α = tumor necrosis factor-α, LBP = lipopolysaccharide-binding protein, CoV = coefficient of variation.

**Table 3:** Available data by visit.

		Kohli		BCPT		1-Back		2-Back	
		Depress by Inflam	Depress by LBP	Depress by Inflam	Depress by LBP	Depress by Inflam	Depress by LBP	Depress by Inflam	Depress by LBP
Study 1	Total	202	202	0	0	0	0	0	0
	Visit 1	199	196	0	0	0	0	0	0
	Visit 2	128	124	0	0	0	0	0	0
	Visit 3	108	106	0	0	0	0	0	0
Study 2	Total	63	56	100	89	0	0	0	0
	Visit 1	52	46	98	88	0	0	0	0
	Visit 2	51	50	88	87	0	0	0	0
	Visit 3	49	47	80	77	0	0	0	0
Study 3	Total	162	161	74	74	0	0	0	0
	Visit 1	85	84	0	0	0	0	0	0
	Visit 2	77	77	74	74	0	0	0	0
Study 4	Total	0	0	148	140	145	137	145	137
	Visit 1	0	0	146	137	143	134	143	134
	Visit 2	0	0	105	103	98	96	97	95
		**Trail-Making Test A**		**Trail-Making Test B**		**Animal-Naming Test and FAS Test**		**CPT**	
		**Depress by Inflam**	**Depress by LBP**	**Depress by Inflam**	**Depress by LBP**	**Depress by Inflam**	**Depress by LBP**	**Depress by Inflam**	**Depress by LBP**
Study 4	Total	147	139	146	138	148	140	148	140
	Visit 1	143	131	140	149	147	138	147	138
	Visit 2	99	97	99	99	99	97	98	96
		**HVLT Recall and Percent Retained**		**HVLT Recognition**					
		**Depress by Inflam**	**Depress by LBP**	**Depress by Inflam**	**Depress by LBP**				
Study 4	Total	148	140	146	139				
	Visit 1	147	138	143	135				
	Visit 2	98	96	98	96				

Depress = depression, Inflam = inflammatory index, LBP = lipopolysaccharide-binding protein; BCPT = Breast Cancer Prevention Trial cognitive functioning; CPT = Conners continuous performance test; HVLT = Hopkins verbal learning test.

**Table 4:** Characteristics of included subjects at first study visit.

		Study 1 (*n* = 202)		Study 2 (*n* = 100)		Study 3 (*n* = 162)		Study 4 (*n* = 149)		Total (*n* = 613)		*p*-Value
Age												<0.0001
	Mean (SD)	55.9	(11.6)	51.3	(8.7)	56.6	(8.6)	52.1	(10.1)	54.4	(10.2)	
	[min, max]	[26, 88]		[28, 76]		[36, 78]		[26, 75]		[26, 88]		
Months since treatment												<0.0001
	Mean (SD)	−5.3	(3.9)	12.1	(8.0)	45.0	(28.3)	−2.3	(4.7)	11.6	(25.9)	
	[min, max]	[−21.4, 0.0]		[2.1, 34.0]		[11.4, 131.3]		[−32.9, 4.6]		[−32.9, 131.3]		
Energy/Fatigue scale, RAND36 *												<0.0001
	Mean (SD)	53.3	(23.4)	44.4	(20.9)	61.0	(19.0)	50.7	(19.9)	53.3	(21.7)	
	[min, max]	[0, 100]		[5, 90]		[0, 95]		[0, 100]		[0, 100]		
BMI												0.20
	Mean (SD)	28.9	(7.3)	27.6	(6.0)	27.7	(5.8)	28.6	(5.7)	28.3	(6.4)	
	[min, max]	[15.8, 58.7]		[16.2, 43.9]		[18.7, 45.5]		[16.8, 49.6]		[15.8, 58.7]		
Race												0.03 ^
	White	79%	(160)	88%	(88)	92%	(149)	86%	(128)	86%	(525)	
	Black	15%	(30)	10%	(10)	6%	(10)	9%	(13)	10%	(63)	
	Asian	3%	(7)	2%	(2)	0.6%	(1)	2%	(3)	2%	(13)	
	Native American	2%	(4)	0%	(0)	0%	(0)	1%	(1)	1%	(5)	
	Other	0%	(1)	0%	(0)	0%	(0)	0%	(0)	0%	(1)	
	Mixed	0%	(0)	0%	(0)	1%	(2)	3%	(4)	1%	(6)	
Education												<0.0001
	HS or less	29%	(58)	7%	(7)	11%	(18)	15%	(22)	17%	(105)	
	Some college	21%	(43)	22%	(22)	17%	(28)	25%	(37)	21%	(130)	
	College grad	24%	(48)	33%	(33)	37%	(60)	31%	(46)	31%	(187)	
	Grad/prof training	26%	(53)	38%	(38)	35%	(56)	30%	(44)	31%	(191)	
Cancer Stage												<0.0001
	Stage 0	18%	(37)	9%	(9)	0%	(0)	0%	(0)	8%	(46)	
	Stage 1	45%	(90)	43%	(43)	47%	(76)	50%	(74)	46%	(283)	
	Stage 2	27%	(55)	38%	(38)	48%	(77)	47%	(70)	39%	(240)	
	Stage 3	9%	(19)	10%	(10)	6%	(9)	3%	(5)	7%	(43)	
	Missing	--	(1)	--	(0)	--	(0)	--	(0)	--	(1)	
Cancer Treatment												<0.0001
	Surgery	30%	(61)	13%	(13)	12%	(19)	31%	(46)	23%	(139)	
	Surgery + chemo	16%	(33)	23%	(23)	27%	(44)	10%	(15)	19%	(115)	
	Surgery + radiation	27%	(55)	24%	(24)	23%	(37)	32%	(47)	27%	(163)	
	Surgery + chemo + radiation	26%	(53)	40%	(40)	38%	(62)	28%	(41)	32%	(196)	
Antidepressant Use												0.41
	No	77%	(155)	70%	(70)	71%	(115)	76%	(113)	74%	(453)	
	Yes	23%	(46)	30%	(30)	29%	(47)	24%	(36)	26%	(159)	
	Missing	--	(1)	--	(0)	--	(0)	--	(0)	--	(1)	

* Missing for 1 participant in Study 4 sample. ^ Tested after collapsing to 3 levels: White, Black, Other.

**Table 5:** Inflammatory markers and depressive symptoms for included subjects across all study visits used in at least one analysis model.

		Study 1 (*n* = 435 Visits)	Study 2 (*n* = 266 Visits)	Study 3 (*n* = 162 Visits)	Study 4 (*n* = 252 Visits)	Total (*n* = 1116 Visits)
IL-6						
	M(SD)	2.2 (2.3)	2.3 (2.2)	2.8 (6.3)	2.9 (2.4)	2.5 (3.2)
	[min, max]	[0.15, 21.8]	[0.15, 15.1]	[0.44, 78.4]	[0.06, 14.0]	[0.06, 78.4]
TNF-α						
	M(SD)	7.4 (3.8)	7.1 (3.2)	2.3 (0.6)	2.4 (0.7)	5.5 (3.7)
	[min, max]	[1.3, 28.4]	[2.1, 27.0]	[1.2, 4.5]	[1.0, 4.7]	[1.0, 28.4]
CRP						
	M(SD)	3.0 (5.2)	2.2 (3.9)	3.2 (4.6)	5.4 (14.6)	3.4 (8.2)
	[min, max]	[0.15, 53.6]	[0.15, 34.0]	[0.10, 29.0]	[0.06, 194.6]	[0.1, 194.6]
LBP						
	M(SD)	5069 (2220)	5562 (1947)	4150 (1918)	5225 (2368)	5082 (2192)
	[min, max]	[221, 14,218]	[442, 10,719]	[429, 10,504]	[890, 17,070]	[221, 17,070]
CES-D						
	M(SD)	13.8 (10.5)	10.2 (8.6)	7.7 (7.1)	9.4 (6.7)	11.1 (9.1)
	[min, max]	[0, 49]	[0, 46]	[0, 41]	[0, 34]	[0, 49]
CES-D clinical cutoff, %(n)						
	<16	62% (270)	79% (211)	87% (141)	83% (208)	74% (830)
	16+	38% (165)	21% (55)	13% (21)	17% (44)	26% (285)

IL-6 = interleukin-6, TNF-α = tumor necrosis factor-α, CRP = C-reactive protein, LBP = lipopolysaccharide-binding protein, CES-D = Center for Epidemiological Studies Depression Scale.

**Table 6:** Outcome values across all study visits used in analyses.

Outcome	Study	N	Mean (SD)		[Min, Max]	
Outcomes in Multiple Studies:						
Kohli Focus						
	Study 1	435	2.30	(2.27)	[0, 9]	
	Study 2	152	3.05	(2.35)	[0, 10]	
	Study 3	162	2.07	(1.99)	[0, 8]	
	All Three Combined	749	2.40	(2.25)	[0, 10]	
Kohli Memory						
	Study 1	435	2.11	(2.24)	[0, 9]	
	Study 2	152	3.36	(2.25)	[0, 10]	
	Study 3	162	2.24	(2.03)	[0, 8]	
	All Three Combined	749	2.39	(2.25)	[0, 10]	
BCPT						
	Study 2	266	1.34	(0.98)	[0, 4]	
	Study 3	74	0.91	(0.71)	[0, 2.7]	
	Study 4	251	1.09	(0.83)	[0, 4]	
	All Three Combined	591	1.18	(0.90)	[0, 4]	
Study 4 Only:						
FAS Test		246	38.2	(11.0)	[10, 67]	
Animal-Naming Test		246	20.1	(4.7)	[7, 35]	
CPT Detectability		245	44.3	(8.3)	[25, 77]	
CPT Omissions		245	46.1	(4.8)	[44, 90]	
CPT Commissions		245	46.3	(8.0)	[34, 86]	
CPT Hit RT		245	48.5	(8.5)	[31, 81]	
HVLT Recall		245	27.0	(4.1)	[15, 35]	
HVLT Percent Retained		245	92.3	(14.9)	[0, 133.3]	
HVLT Recognition		241	11.6	(0.84)	[7, 12]	
1-back Accuracy		241	0.95	(0.07)	[0.47, 1]	
1-back RT		241	630	(113)	[371, 1120]	
2-back Accuracy		240	0.87	(0.12)	[0.30, 0.98]	
2-back RT		240	782	(124)	[473, 1083]	
Trail A Seconds to Complete		242	26.9	(9.0)	[13, 81]	
Trail B Seconds to Complete		239	65.5	(28.5)	[30, 260]	

BCPT = Breast Cancer Prevention Trial cognitive functioning; CPT = Conners continuous performance test; HVLT = Hopkins verbal learning test.

**Table 7:** Results summary.

		Depression by Inflammation	Depression by LBP	Inflammation Main Effect	LBP Main Effect	Depression Main Effect
**Subjective**	Kohli focus	X	X			X
	Kohli memory	marginal	marginal			X
	BCPT					X
**Objective**	FAS number					X
	Animal number			marginal		marginal
	CPT detectability					X
	CPT omissions					
	CPT commissions					X
	CPT Hit RT					
	Hopkins recall total	X (in opposite direction)	marginal (in opposite direction)			X
	Hopkins retain percent				marginal	
	Hopkins recognition				marginal	
	1-back accuracy					
	1-back RT					
	2-back accuracy			marginal		X
	2-back RT				X	
	Trail Making Test A Seconds to Complete		marginal		X	marginal
	Trail Making Test B Seconds to Complete					X
	KEY:					
	Verbal Fluency					
	Attention					
	Verbal Learning and Memory					
	Working Memory					
	Visuospatial Search					

RT = response time, CPT = Conners continuous performance test, BCPT = Breast Cancer Prevention Trial Cognitive Subscale, LBP = lipopolysaccharide-binding protein. Note that the depression main effects and inflammation main effects come from the inflammation by depression moderation models, and the LBP main effect comes from the LBP by depression moderation models.

## Data Availability

The data presented in this study are openly available in Open Science Forum at https://osf.io/m5zrq/?view_only=eb62317f6bdf4031abc84b6988e38fb1.

## Supplementary Materials

The following supporting information can be downloaded at: https://www.mdpi.com/article/10.3390/cancers15174414/s1, Table S1: Correlations between study variables Table S2: Results from models that do not adjust for BMI and fatigue.

## References

[1] Joly F., Lange M., Dos Santos M., Vaz-Luis I., Di Meglio A. (2019). Long-Term Fatigue and Cognitive Disorders in Breast Cancer Survivors. Cancers.

[2] Dijkshoorn A.B., van Stralen H.E., Sloots M., Schagen S.B., Visser-Meily J.M., Schepers V.P. (2021). Prevalence of Cognitive Impairment and Change in Patients with Breast Cancer: A Systematic Review of Longitudinal Studies. Psycho-Oncology.

[3] Bernstein L.J., McCreath G.A., Komeylian Z., Rich J.B. (2017). Cognitive Impairment in Breast Cancer Survivors Treated with Chemotherapy Depends on Control Group Type and Cognitive Domains Assessed: A Multilevel Meta-Analysis. Neurosci. Biobehav. Rev..

[4] Whittaker A.L., George R.P., O’Malley L. (2022). Prevalence of Cognitive Impairment Following Chemotherapy Treatment for Breast Cancer: A Systematic Review and Meta-Analysis. Sci. Rep..

[5] Boscher C., Joly F., Clarisse B., Humbert X., Grellard J.-M., Binarelli G., Tron L., Licaj I., Lange M. (2020). Perceived Cognitive Impairment in Breast Cancer Survivors and Its Relationships with Psychological Factors. Cancers.

[6] Hutchinson A.D., Hosking J.R., Kichenadasse G., Mattiske J.K., Wilson C. (2012). Objective and Subjective Cognitive Impairment Following Chemotherapy for Cancer: A Systematic Review. Cancer Treat. Rev..

[7] Pullens M.J., De Vries J., Roukema J.A. (2010). Subjective Cognitive Dysfunction in Breast Cancer Patients: A Systematic Review. Psycho-Oncology.

[8] Von Ah D., Habermann B., Carpenter J.S., Schneider B.L. (2013). Impact of Perceived Cognitive Impairment in Breast Cancer Survivors. Eur. J. Oncol. Nurs..

[9] Savard J., Ganz P.A. (2016). Subjective or Objective Measures of Cognitive Functioning—What’s More Important?. JAMA Oncol..

[10] Kohli S., Griggs J.J., Roscoe J.A., Jean-Pierre P., Bole C., Mustian K.M., Hill R., Smith K., Gross H., Morrow G.R. (2007). Self-Reported Cognitive Impairment in Patients with Cancer. J. Oncol. Pract..

[11] Von Ah D., Tallman E.F. (2015). Perceived Cognitive Function in Breast Cancer Survivors: Evaluating Relationships with Objective Cognitive Performance and Other Symptoms Using the Functional Assessment of Cancer Therapy—Cognitive Function Instrument. J. Pain Symptom Manag..

[12] Charles C., Bardet A., Larive A., Gorwood P., Ramoz N., Thomas E., Viari A., Rousseau-Tsangaris M., Dumas A., Menvielle G. (2022). Characterization of Depressive Symptoms Trajectories after Breast Cancer Diagnosis in Women in France. JAMA Netw. Open.

[13] Rock P.L., Roiser J.P., Riedel W.J., Blackwell A. (2014). Cognitive Impairment in Depression: A Systematic Review and Meta-Analysis. Psychol. Med..

[14] Seliktar N., Polek C., Brooks A., Hardie T. (2015). Cognition in Breast Cancer Survivors: Hormones versus Depression. Psycho-Oncology.

[15] Cacioppo J.T., Hawkley L.C., Thisted R.A. (2010). Perceived Social Isolation Makes Me Sad: 5-Year Cross-Lagged Analyses of Loneliness and Depressive Symptomatology in the Chicago Health, Aging, and Social Relations Study. Psychol. Aging.

[16] Jaremka L.M., Peng J., Bornstein R., Alfano C.M., Andridge R.R., Povoski S.P., Lipari A.M., Agnese D.M., Farrar W.B., Yee L.D. (2014). Cognitive Problems among Breast Cancer Survivors: Loneliness Enhances Risk. Psycho-Oncology.

[17] American Psychiatric Association D., American Psychiatric Association (2013). Diagnostic and Statistical Manual of Mental Disorders: DSM-5.

[18] Zimmerman M., Ellison W., Young D., Chelminski I., Dalrymple K. (2015). How Many Different Ways Do Patients Meet the Diagnostic Criteria for Major Depressive Disorder?. Compr. Psychiatry.

[19] Osimo E.F., Baxter L.J., Lewis G., Jones P.B., Khandaker G.M. (2019). Prevalence of Low-Grade Inflammation in Depression: A Systematic Review and Meta-Analysis of CRP Levels. Psychol. Med..

[20] Mac Giollabhui N., Ng T.H., Ellman L.M., Alloy L.B. (2020). The Longitudinal Associations of Inflammatory Biomarkers and Depression Revisited: Systematic Review, Meta-Analysis, and Meta-Regression. Mol. Psychiatry.

[21] Chang H.H., Lee I.H., Gean P.W., Lee S.-Y., Chi M.H., Yang Y.K., Lu R.-B., Chen P.S. (2012). Treatment Response and Cognitive Impairment in Major Depression: Association with C-Reactive Protein. Brain Behav. Immun..

[22] Carlier A., Rhebergen D., Veerhuis R., Schouws S., Oudega M.L., Eikelenboom P., Bouckaert F., Sienaert P., Obbels J., Stek M.L. (2021). Inflammation and Cognitive Functioning in Depressed Older Adults Treated with Electroconvulsive Therapy: A Prospective Cohort Study. J. Clin. Psychiatry.

[23] Krogh J., Benros M.E., Jørgensen M.B., Vesterager L., Elfving B., Nordentoft M. (2014). The Association between Depressive Symptoms, Cognitive Function, and Inflammation in Major Depression. Brain Behav. Immun..

[24] Mac Giollabhui N., Alloy L.B., Schweren L.J.S., Hartman C.A. (2021). Investigating Whether a Combination of Higher CRP and Depression Is Differentially Associated with Worse Executive Functioning in a Cohort of 43,896 Adults. Brain Behav. Immun..

[25] Villasenor A., Flatt S.W., Marinac C., Natarajan L., Pierce J.P., Patterson R.E. (2014). Postdiagnosis C-Reactive Protein and Breast Cancer Survivorship: Findings from the WHEL StudyC-Reactive Protein and Long-Term Breast Cancer Survival. Cancer Epidemiol. Biomark. Prev..

[26] Carroll J.E., Nakamura Z.M., Small B.J., Zhou X., Cohen H.J., Ahles T.A., Ahn J., Bethea T.N., Extermann M., Graham D. (2023). Elevated C-Reactive Protein and Subsequent Patient-Reported Cognitive Problems in Older Breast Cancer Survivors: The Thinking and Living with Cancer Study. J. Clin. Oncol..

[27] Camilleri M. (2019). Leaky Gut: Mechanisms, Measurement and Clinical Implications in Humans. Gut.

[28] Madison A.A., Andridge R., Padin A.C., Wilson S., Bailey M.T., Alfano C.M., Povoski S.P., Lipari A.M., Agnese D.M., Carson W.E. (2020). Endotoxemia Coupled with Heightened Inflammation Predicts Future Depressive Symptoms. Psychoneuroendocrinology.

[29] Madison A.A., Belury M.A., Andridge R., Shrout M.R., Renna M.E., Malarkey W.B., Bailey M.T., Kiecolt-Glaser J.K. (2020). Afternoon Distraction: A High-Saturated-Fat Meal and Endotoxemia Impact Postmeal Attention in a Randomized Crossover Trial. Am. J. Clin. Nutr..

[30] Moreno-Navarrete J., Blasco G., Puig J., Biarnés C., Rivero M., Gich J., Fernandez-Aranda F., Garre-Olmo J., Ramio-Torrenta L., Alberich-Bayarri A. (2017). Neuroinflammation in Obesity: Circulating Lipopolysaccharide-Binding Protein Associates with Brain Structure and Cognitive Performance. Int. J. Obes..

[31] Kiecolt-Glaser J.K., Bennett J.M., Andridge R., Peng J., Shapiro C.L., Malarkey W.B., Emery C.F., Layman R., Mrozek E.E., Glaser R. (2014). Yoga’s Impact on Inflammation, Mood, and Fatigue in Breast Cancer Survivors: A Randomized Controlled Trial. J. Clin. Oncol..

[32] Kiecolt-Glaser J.K., Renna M.E., Peng J., Sheridan J., Lustberg M., Ramaswamy B., Wesoloski R., VanDeusen J.B., Williams N.O., Sardesai S.D. (2022). Breast Cancer Survivors’ Vaccine Responses: Chemotherapy, Obesity, and Fitness Make a Difference. Brain Behav. Immun..

[33] Brydon L., Harrison N.A., Walker C., Steptoe A., Critchley H.D. (2008). Peripheral Inflammation Is Associated with Altered Substantia Nigra Activity and Psychomotor Slowing in Humans. Biol. Psychiatry.

[34] Madison A.A., Filatov M., Andridge R., Haas G., Povoski S.P., Agnese D.M., Lustberg M., Reinbolt R.E., Wesolowski R., Williams N.O. (2023). A Troubled Heart: Mood Disorder History Longitudinally Predicts Faster Cardiopulmonary Aging in Breast Cancer Survivorship. PLoS ONE.

[35] Terhorst L., Blair-Belansky H., Moore P.J., Bender C. (2011). Evaluation of the Psychometric Properties of the BCPT Symptom Checklist with a Sample of Breast Cancer Patients before and after Adjuvant Therapy. Psycho-Oncology.

[36] Lin Z., Tam F., Churchill N.W., Lin F.-H., MacIntosh B.J., Schweizer T.A., Graham S.J. (2021). Trail Making Test Performance Using a Touch-Sensitive Tablet: Behavioral Kinematics and Electroencephalography. Front. Hum. Neurosci..

[37] Kortte K.B., Horner M.D., Windham W.K. (2002). The Trail Making Test, Part B: Cognitive Flexibility or Ability to Maintain Set?. Appl. Neuropsychol..

[38] Malek-Ahmadi M., Small B.J., Raj A. (2012). The Diagnostic Value of Controlled Oral Word Association Test-FAS and Category Fluency in Single-Domain Amnestic Mild Cognitive Impairment. Dement. Geriatr. Cogn. Disord..

[39] Campagna F., Montagnese S., Ridola L., Senzolo M., Schiff S., De Rui M., Pasquale C., Nardelli S., Pentassuglio I., Merkel C. (2017). The Animal Naming Test: An Easy Tool for the Assessment of Hepatic Encephalopathy. Hepatology.

[40] Conners C.K. (2014). Conners Continuous Performance Test 3rd Edition (Conners CPT 3) & Connors Continuous Auditory Test of Attention (Conners CATA): Technical Manual.

[41] Scimeca L.M., Holbrook L., Rhoads T., Cerny B.M., Jennette K.J., Resch Z.J., Obolsky M.A., Ovsiew G.P., Soble J.R. (2021). Examining Conners Continuous Performance Test-3 (CPT-3) Embedded Performance Validity Indicators in an Adult Clinical Sample Referred for ADHD Evaluation. Dev. Neuropsychol..

[42] Rabin L.A., Barr W.B., Burton L.A. (2005). Assessment Practices of Clinical Neuropsychologists in the United States and Canada: A Survey of INS, NAN, and APA Division 40 Members. Arch. Clin. Neuropsychol..

[43] Benedict R.H.B., Schretlen D., Groninger L., Brandt J. (1998). Hopkins Verbal Learning Test–Revised: Normative Data and Analysis of Inter-Form and Test-Retest Reliability. Clin. Neuropsychol..

[44] Chiaravalloti N.D., DeLuca J., Moore N.B., Ricker J.H. (2005). Treating Learning Impairments Improves Memory Performance in Multiple Sclerosis: A Randomized Clinical Trial. Mult. Scler. J..

[45] Kane M.J., Conway A.R.A., Miura T.K., Colflesh G.J.H. (2007). Working Memory, Attention Control, and the N-Back Task: A Question of Construct Validity. J. Exp. Psychol. Learn. Mem. Cogn..

[46] Kirchner W.K. (1958). Age Differences in Short-Term Retention of Rapidly Changing Information. J. Exp. Psychol..

[47] Radloff L.S. (1977). The CES-D Scale: A Self-Report Depression Scale for Research in the General Population. Appl. Psychol. Meas..

[48] Weissman M.M., Sholomskas D., Pottenger M., Prusoff B.A., Locke B.Z. (1977). Assessing Depressive Symptoms in Five Psychiatric Populations: A Validation Study. Am. J. Epidemiol..

[49] Fontes J.D., Yamamoto J.F., Larson M.G., Wang N., Dallmeier D., Rienstra M., Schnabel R.B., Vasan R.S., Keaney Jr J.F., Benjamin E.J. (2013). Clinical Correlates of Change in Inflammatory Biomarkers: The Framingham Heart Study. Atherosclerosis.

[50] Kell D.B., Pretorius E. (2015). On the Translocation of Bacteria and Their Lipopolysaccharides between Blood and Peripheral Locations in Chronic, Inflammatory Diseases: The Central Roles of LPS and LPS-Induced Cell Death. Integr. Biol..

[51] Brenchley J.M., Price D.A., Schacker T.W., Asher T.E., Silvestri G., Rao S., Kazzaz Z., Bornstein E., Lambotte O., Altmann D. (2006). Microbial Translocation Is a Cause of Systemic Immune Activation in Chronic HIV Infection. Nat. Med..

[52] Gonzalez-Quintela A., Alonso M., Campos J., Vizcaino L., Loidi L., Gude F. (2013). Determinants of Serum Concentrations of Lipopolysaccharide-Binding Protein (LBP) in the Adult Population: The Role of Obesity. PLoS ONE.

[53] Abad-Fernández M., Vallejo A., Hernández-Novoa B., Díaz L., Gutiérrez C., Madrid N., Muñoz M.Á., Moreno S. (2013). Correlation Between Different Methods to Measure Microbial Translocation and Its Association with Immune Activation in Long-Term Suppressed HIV-1–Infected Individuals. JAIDS J. Acquir. Immune Defic. Syndr..

[54] Uhde M., Ajamian M., Caio G., De Giorgio R., Indart A., Green P.H., Verna E.C., Volta U., Alaedini A. (2016). Intestinal Cell Damage and Systemic Immune Activation in Individuals Reporting Sensitivity to Wheat in the Absence of Coeliac Disease. Gut.

[55] Tremellen K., Pearce K. (2020). Small Intestinal Bacterial Overgrowth (SIBO) as a Potential Cause of Impaired Spermatogenesis. Gut.

[56] Lakatos P.L., Kiss L.S., Palatka K., Altorjay I., Antal-Szalmas P., Palyu E., Udvardy M., Molnar T., Farkas K., Veres G. (2010). Serum Lipopolysaccharide-Binding Protein and Soluble CD14 Are Markers of Disease Activity in Patients with Crohn’s Disease. Inflamm. Bowel Dis..

[57] Vander Zee K.I., Sanderman R., Heyink J.W., de Haes H. (1996). Psychometric Qualities of the RAND 36-Item Health Survey 1.0: A Multidimensional Measure of General Health Status. Int. J. Behav. Med..

[58] Walker K.A., Gottesman R.F., Wu A., Knopman D.S., Gross A.L., Mosley T.H., Selvin E., Windham B.G. (2019). Systemic Inflammation during Midlife and Cognitive Change over 20 Years: The ARIC Study. Neurology.

[59] Mac Giollabhui N., Ellman L.M., Coe C.L., Byrne M.L., Abramson L.Y., Alloy L.B. (2020). To Exclude or Not to Exclude: Considerations and Recommendations for C-Reactive Protein Values Higher than 10 Mg/L. Brain Behav. Immun..

[60] Ballinger G.A. (2004). Using Generalized Estimating Equations for Longitudinal Data Analysis. Organ. Res. Methods.

[61] Zeger S.L., Liang K.-Y. (1986). Longitudinal Data Analysis for Discrete and Continuous Outcomes. Biometrics.

[62] Capuron L., Gumnick J.F., Musselman D.L., Lawson D.H., Reemsnyder A., Nemeroff C.B., Miller A.H. (2002). Neurobehavioral Effects of Interferon-α in Cancer Patients: Phenomenology and Paroxetine Responsiveness of Symptom Dimensions. Neuropsychopharmacology.

[63] Sundermann E.E., Biegon A., Rubin L.H., Lipton R.B., Landau S., Maki P.M., Alzheimer’s Disease Neuroimaging Initiative (2017). Does the Female Advantage in Verbal Memory Contribute to Underestimating Alzheimer’s Disease Pathology in Women versus Men?. J. Alzheimer’s Dis..

[64] Oliver M.D., Morrison C., Kamal F., Graham J., Dadar M. (2022). Subjective Cognitive Decline Is a Better Marker for Future Cognitive Decline in Females than in Males. Alzheimer’s Res. Ther..

[65] Ahmadi S., Razazan A., Nagpal R., Jain S., Wang B., Mishra S.P., Wang S., Justice J., Ding J., McClain D.A. (2020). Metformin Reduces Aging-Related Leaky Gut and Improves Cognitive Function by Beneficially Modulating Gut Microbiome/Goblet Cell/Mucin Axis. J. Gerontol. Ser. A.

[66] Wang X., Liu G., Gao Q., Li N., Wang R. (2020). C-type Lectin-like Receptor 2 and Zonulin Are Associated with Mild Cognitive Impairment and Alzheimer’s Disease. Acta Neurol. Scand..

[67] Ryan L.A., Zheng J., Brester M., Bohac D., Hahn F., Anderson J., Ratanasuwan W., Gendelman H.E., Swindells S. (2001). Plasma Levels of Soluble CD14 and Tumor Necrosis Factor-α Type II Receptor Correlate with Cognitive Dysfunction during Human Immunodeficiency Virus Type 1 Infection. J. Infect. Dis..

[68] Lyons J.L., Uno H., Ancuta P., Kamat A., Moore D.J., Singer E.J., Morgello S., Gabuzda D. (2011). Plasma SCD14 Is a Biomarker Associated with Impaired Neurocognitive Test Performance in Attention and Learning Domains in HIV Infection. J. Acquir. Immune Defic. Syndr..

[69] Rundek T., Roy S., Hornig M., Cheung Y.K., Gardener H., DeRosa J., Levin B., Wright C.B., Del Brutto V.J., Elkind M.S. (2021). Gut Permeability and Cognitive Decline: A Pilot Investigation in the Northern Manhattan Study. Brain Behav. Immun.-Health.

[70] Ufnal M., Pham K. (2017). The Gut-Blood Barrier Permeability—A New Marker in Cardiovascular and Metabolic Diseases?. Med. Hypotheses.

[71] Zanoli L., Briet M., Empana J.P., Cunha P.G., Mäki-Petäjä K.M., Protogerou A.D., Tedgui A., Touyz R.M., Schiffrin E., Spronck B. (2020). Vascular Consequences of Inflammation: A Position Statement from the ESH Working Group on Vascular Structure and Function and the ARTERY Society. J. Hypertens..

[72] Bradshaw P.T., Stevens J., Khankari N., Teitelbaum S.L., Neugut A.I., Gammon M.D. (2016). Cardiovascular Disease Mortality among Breast Cancer Survivors. Epidemiology.

[73] Felger J.C., Haroon E., Patel T.A., Goldsmith D.R., Wommack E.C., Woolwine B.J., Le N.-A., Feinberg R., Tansey M.G., Miller A.H. (2018). What Does Plasma CRP Tell Us about Peripheral and Central Inflammation in Depression?. Mol. Psychiatry.

[74] McAfoose J., Baune B.T. (2009). Evidence for a Cytokine Model of Cognitive Function. Neurosci. Biobehav. Rev..

[75] Turkheimer F.E., Veronese M., Mondelli V., Cash D., Pariante C.M. (2023). Sickness Behaviour and Depression: An Updated Model of Peripheral-Central Immunity Interactions. Brain Behav. Immun..

[76] Derry H.M., Padin A.C., Kuo J.L., Hughes S., Kiecolt-Glaser J.K. (2015). Sex Differences in Depression: Does Inflammation Play a Role?. Curr. Psychiatry Rep..

[77] Wang L., Xue Y., Cao S., Xie Y., Wu C., Ruffaner-Hanson C.D., Tang H., Teng Z., Chen J., Tang M. (2020). Sex Differences in the Cognitive Function of First-Diagnosed, Drug-Naïve Depressed Patients: An Observational Case-Control Study. J. Affect. Disord..

[78] Kiecolt-Glaser J.K., Belury M.A., Andridge R., Malarkey W.B., Hwang B.S., Glaser R. (2012). Omega-3 Supplementation Lowers Inflammation in Healthy Middle-Aged and Older Adults: A Randomized Controlled Trial. Brain Behav. Immun..

[79] Madison A.A., Belury M.A., Andridge R., Renna M.E., Shrout M.R., Malarkey W.B., Lin J., Epel E.S., Kiecolt-Glaser J.K. (2021). Omega-3 Supplementation and Stress Reactivity of Cellular Aging Biomarkers: An Ancillary Substudy of a Randomized, Controlled Trial in Midlife Adults. Mol. Psychiatry.

[80] Kaliannan K., Wang B., Li X.-Y., Kim K.-J., Kang J.X. (2015). A Host-Microbiome Interaction Mediates the Opposing Effects of Omega-6 and Omega-3 Fatty Acids on Metabolic Endotoxemia. Sci. Rep..

[81] Barnes S., Chowdhury S., Gatto N.M., Fraser G.E., Lee G.J. (2021). Omega-3 Fatty Acids Are Associated with Blood–Brain Barrier Integrity in a Healthy Aging Population. Brain Behav..

